# Trends of Ovarian Cancer Incidence by Histotype and Race/Ethnicity in the United States 1992–2019

**DOI:** 10.1158/2767-9764.CRC-22-0410

**Published:** 2023-01-03

**Authors:** Minh Tung Phung, Celeste Leigh Pearce, Rafael Meza, Jihyoun Jeon

**Affiliations:** 1 Department of Epidemiology, University of Michigan, Ann Arbor, Michigan.; 2 Department of Integrative Oncology, BC Cancer Research Institute, Vancouver, British Columbia, Canada.

## Abstract

The effect of risk factors on ovarian cancer differs by histotype, and the prevalence of such risk factors varies by race/ethnicity. It is not clear how ovarian cancer incidence has changed over time by histotype and race/ethnicity.

We used the Surveillance, Epidemiology, and End Results Program (SEER-12) 1992–2019 data to examine the trend of ovarian cancer incidence for three histotypes (high-grade serous *N* = 19,691, endometrioid *N* = 3,212, and clear cell *N* = 3,275) and four racial/ethnic groups (Asian/Pacific Islander, Hispanic, non-Hispanic Black, and non-Hispanic White). Joinpoint and age-period-cohort analyses were conducted to analyze ovarian cancer incidence trends.

High-grade serous cancer was the most common histotype, but its incidence has significantly decreased over time for all racial/ethnic groups; the decrease was largest for non-Hispanic White women (average annual percent change AAPC during 2010–2019 = −6.1; 95% confidence interval (CI), −8.0 to −4.2). Conversely, clear cell cancer was most common in the Asian/Pacific Islanders, and its incidence has increased over time, particularly among Hispanic and Asian/Pacific Islander women (AAPC during 2010–2019 = 2.8; 95% CI, 0.8 to 4.7, and AAPC = 1.5; 95% CI, 0.7 to 2.2, respectively). Endometrioid cancer incidence has decreased in non-Hispanic White but increased in Hispanic women (AAPC during 2010–2019 = −1.3; 95% CI, −1.9 to −0.8, and AAPC = 3.6; 95% CI, 1.0 to 6.3, respectively).

The differential incidence trends by histotype and race/ethnicity underscore the need to monitor incidence and risk factor trends across different groups and develop targeted preventive interventions to reduce the burden of ovarian cancer and disparity by race/ethnicity.

**Significance:**

During 1992–2019, high-grade serous ovarian cancer incidence has decreased while clear cell cancer incidence has increased regardless of race/ethnicity. Endometrioid cancer incidence has decreased in non-Hispanic White but increased in Hispanic women. Differential ovarian cancer incidence trends highlight the need for targeted preventive interventions by histotype and race/ethnicity.

## Introduction

Approximately 22,000 women in the United States are diagnosed with ovarian cancer annually (1), equating to a lifetime risk of approximately 1.3% (2). Survival is low: ovarian cancer is the fifth leading cause of cancer deaths for women in the United States, and the 5-year survival rate is less than 50% (1, 3). Coburn and colleagues demonstrated that in both White and Black women, the U.S. rates of ovarian cancer decreased from 1983–1987 through 2003–2007 based on data from Cancer Incidence in Five Continents (CI5; ref. 4).

The declines in ovarian cancer rates have been mainly attributed to increasing parity during the 1940s to 1960s baby boom (5) and the introduction of combined oral contraceptives in the 1960s (6). A woman's risk of ovarian cancer has been shown to decrease as a function of parity (7–12), and the number of births per woman in the United States has also been declining in recent decades (5). Similarly, combined oral contraceptive use is an unequivocal protective factor for ovarian cancer, and 5 years of use is associated with an approximate 50% decreased risk of the disease (9). However, there has been a dramatic change in combined oral contraceptive use, with the prevalence declining among 15–44 year olds during 2000–2016 (13). This decrease in combined oral contraceptive use is likely to continue given the increasing popularity of progestin-releasing intrauterine devices (13). In contrast, estrogen–alone menopausal hormonal therapy (ET) use is associated with an increased risk of serous and endometrioid ovarian cancers by approximately 60% (14, 15). Use of ET decreased following the findings of the Women's Health Initiative (WHI; ref. 16). Thus, the prevalence of protective and risk factors for the disease is changing over time, which may impact ovarian cancer incidence trends.

Notably, there has been a sea change in our understanding of ovarian cancer over the past two decades. Ovarian cancer is now largely thought of as five distinct histotypes: high-grade serous, low-grade serous, endometrioid, clear cell, and mucinous, depending on different cells of origin. Many high-grade serous cancers likely arise in the fallopian tube fimbriae, whereas endometriosis is widely considered the tissue of origin for clear cell and endometrioid cancers (17). Many risk factors for these histotypes are shared, including parity and combined oral contraceptive use, but some are distinct by histotype. As mentioned above, ET use is not associated with the risk of clear cell or mucinous histotypes (14). On the basis of data from CI5 among White women in the United States, there have been decreases in endometrioid and mucinous ovarian cancers during 1988–2007. Still, ovarian cancer rates of serous and clear cell have been relatively constant (4), which was not fully understood.

Ovarian cancer incidence is highest among non-Hispanic White women and lowest among non-Hispanic Black and Asian/Pacific Islander women (2). Wu and colleagues suggested that much of the difference in incidence by race/ethnicity could be explained by the distribution of underlying risk and protective factors for the disease (18).

It is crucial to analyze the trends of ovarian cancer incidence by histotype and race/ethnicity as the risk factors vary by histotype and the prevalence of risk factors differs by race/ethnicity. Some previous studies have examined the trends of ovarian cancer in the United States. However, limitations of these studies include not evaluating the trends by histotypes (19), and restricting to Black and White women (4, 19–21). In addition, we extended the previous ovarian trend analysis based on the Surveillance, Epidemiology, and End Results (SEER) Program (22), using the recently updated data until 2019.

To understand both historical and current trends in ovarian cancer rates and to overcome the limitations of the previous studies, we have conducted Joinpoint regression and age-period-cohort analyses using the most recent data from SEER during 1992–2019 (22). We evaluated incidence trend across histotypes and racial/ethnic groups (Asian/Pacific Islander, Hispanic, non-Hispanic Black, and non-Hispanic White) to fully understand the state of ovarian cancer risk in the United States.

## Materials and Methods

All analyses are based on publicly available data; therefore, Institutional Review Board approval was not required.

### Data

Data for ovarian cancer cases and population denominators were obtained from the SEER-12 registry November 2021 submission using SEER*Stat software (version 8.4.0; ref. 23). SEER-12 covers the period from 1992 to 2019. SEER-12 (previously SEER-13) included data from 12 registries (Supplementary Table S1); data from the Detroit registry since 2019 were no longer included in the SEER database. SEER-12 data, rather than SEER-8 (previously SEER-9) or SEER-17 (previously SEER-18), were used because they cover all years during which the Asian/Pacific Islander category was included in the SEER database.

Epithelial ovarian cancer cases meeting the following criteria were extracted from the SEER-12 registry: microscopically confirmed invasive ovarian cancer (primary site: C56.9 ovary), diagnosed between 1992 and 2019, and ages 30–84 years. We excluded women younger than 30 due to the very low incidence of ovarian cancer in this age group (2). Histotypes were classified on the basis of the ICD-O-3 morphology codes, including serous (8050, 8120–8122, 8050, 8120, 8130, 8260, 8441, 8442, 8450, 8460–8463, 9014), endometrioid (8380–8383, 8482, 8570), clear cell (8290, 8310, 8313, 8443, 8444), and mucinous (8470–8472, 8480, 8481, 9015) as described in Peres and colleagues (24). We focused on three main histotypes: high-grade serous, endometrioid, and clear cell, as their etiologies are well understood, they represent the majority of ovarian cancers, and the sample sizes were adequate for our analytic approach. Although the number of mucinous ovarian cancer cases is comparable with clear cell, we did not include mucinous cancer in the analysis because a majority of mucinous ovarian cancers are considered misclassified gastrointestinal primary tumors (25). Serous grade 2, 3, or 4 and endometrioid grade 3 or 4 were classified as high-grade serous cancer as suggested by the literature (24, 26); endometroid grade 1 or 2 were classified as low-grade endometroid cancer. Serous and endometrioid cancers with missing grades (*N* = 5,313 and *N* = 582, respectively) were excluded from the main analysis. All clear cell cancers were considered as such regardless of their grade. Our study included 19,691 high-grade serous, 3,212 low-grade endometrioid, and 3,275 clear cell ovarian cancer cases from the SEER-12 registry. The flow chart of inclusion is presented in Supplementary Fig. S1.

### Age-standardized Incidence Rates

Age-standardized incidence rates (ASIR) for invasive epithelial ovarian cancer for each histotype (high-grade serous, low-grade endometrioid, and clear cell) were calculated separately for four racial/ethnic groups (Asian/Pacific Islander, Hispanic, non-Hispanic Black, and non-Hispanic White). Other racial/ethnic groups were excluded because of the small sample size (Supplementary Fig. S1). ASIRs were standardized to the 2000 U.S. population (27).

### Joinpoint Trend Analysis

Trend analyses were performed using Joinpoint Regression Program software (version 4.9.1.0; ref. 28). Piecewise-continuous log-linear models by histotype and race/ethnicity were fitted, allowing a maximum of five Joinpoints per model. The final models were selected using permutation tests (29). The method used 4,499 permutation tests to ensure that the probability of overall type 1 error was less than 0.05 (29). Annual percent change (APC) in incidence, average annual percent change (AAPC) for the most recent 5 years (2015–2019) and 10 years (2010–2019), and 95% confidence intervals (CI) were calculated.

### Analysis of Incidence by Age-period-cohort

Age-period-cohort analyses were performed by fitting a log-linear model with a Poisson distribution to the observed data to estimate age, period, and cohort effects. To address the well-known nonidentifiability problem of age-period-cohort models, we fitted models with either cohort or period constrained to be zero on average with a slope equal to zero. The final selected model was the one with the lowest Akaike information criterion (AIC). Age-period-cohort analyses were conducted for women ages 30–84 by histotype and race/ethnicity. Age, period, and cohort effects were modeled with natural splines. The birth cohort 1935 and the calendar period 2010 were the reference groups. The analyses were conducted using the *Epi* package in R (version 4.2.0).

### Sensitivity Analyses

In the SEER-12 registry dataset, there were 5,092 patients with serous ovarian cancer with missing grade, but stage data available, among whom 4,725 were at the distant or regional stage. Most distant or regional stage cases are high grade. We, therefore, conducted a sensitivity analysis including these distant or regional stage cases with a missing grade as high-grade serous cases to increase the sample size.

In the main analysis above, we included high-grade endometrioid cancer into high-grade serous cancer as the literature suggests that the majority of high-grade endometrioid cancers are high-grade serous cancers. However, we acknowledge that some true high-grade endometrioid cancers exist. Thus, we also conducted a sensitivity analysis in which high-grade endometrioid cancer was not included as high-grade serous cancer.

We also conducted a sensitivity analysis using SEER-17 data (previously SEER-18), which includes more registries but a shorter time frame compared with SEER-12. Sensitivity analyses using the SEER-17 registry included women diagnosed between 2000 and 2019 with the same eligibility criteria described above for SEER-12 analysis. Although the period of SEER-17 is shorter than SEER-12, the number of cases is greater for every histotype and racial/ethnic group because it includes more registries. The Joinpoint analyses using the SEER-17 registry set a maximum of three Joinpoints due to the shorter study period (2000–2019).

### Data Availability

The data analyzed in this study were obtained from the SEER-12 registry November 2021 submission using SEER*Stat software (version 8.4.0; ref. 23).

## Results

### Age-standardized Incidence Trends

Table 1 presents the ASIRs of invasive epithelial ovarian cancer in women aged 30–84 years diagnosed during 1992–2019 in the SEER-12 registry by histotype and race/ethnicity. During 1992–2019, the ASIRs for high-grade serous cancer were higher than those for low-grade endometrioid and clear cell cancers in all racial/ethnic groups (Table 1)**.** Among non-Hispanic White women, the ASIR for high-grade serous cancer was about six times higher than for low-grade endometrioid and clear cell cancers. Non-Hispanic White women had the highest ASIRs for high-grade serous and low-grade endometrioid cancers, while Asian/Pacific Islander women had the highest ASIR for clear cell cancer. Non-Hispanic Black women had the lowest ASIRs for all three histotypes (Table 1).

**TABLE 1:** ASIR (per 1,000,000) of ovarian cancer by histotype and race/ethnicity, SEER-12, 1992–2019

Histotype	Race/ethnicity	Cases	Population	ASIR^a^
High-grade serous	Asian/Pacific Islander	1,929	40,978,520	47.1
	Hispanic	2,497	52,863,602	55.9
	Non-Hispanic Black	1,008	26,288,993	40.7
	Non-Hispanic White	14,257	170,216,893	74.5
Low-grade endometrioid	Asian/Pacific Islander	466	40,978,520	11.5
	Hispanic	440	52,863,602	8.9
	Non-Hispanic Black	134	26,288,993	5.2
	Non-Hispanic White	2,172	170,216,893	12.2
Clear cell	Asian/Pacific Islander	770	40,978,520	18.8
	Hispanic	388	52,863,602	8.0
	Non-Hispanic Black	110	26,288,993	4.3
	Non-Hispanic White	2,007	170,216,893	11.0

^a^ASIR: Age-standardized incidence rate per 1,000,000 based on the 2000 U.S. standard population.

**FIGURE 1:**
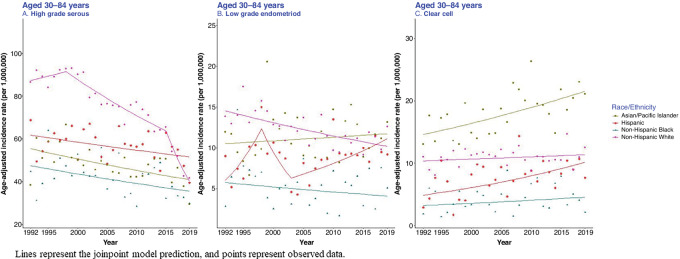
ASIR (per 1,000,000) of ovarian cancer by histotype and race/ethnicity, SEER-12, 1992–2019. Note that the *y*-axis scales are different across the panels.

Although all racial/ethnic groups had a statistically significantly decreasing trend in the ASIRs for high-grade serous cancer in recent years, the decrease was most remarkable for non-Hispanic White women (AAPC for years 2010–2019 = −6.1; 95% CI, −8.0 to −4.2, compared with AAPC = −1.1; 95% CI, −1.8 to −0.5 for Asian/Pacific Islanders, AAPC = −0.7; 95% CI, −1.2 to −0.1 for Hispanic, and AAPC = −1.1; 95% CI, −1.9 to −0.2 for non-Hispanic Black women; Fig. 1 and Table 2). There was a significantly decreasing trend in the ASIRs for low-grade endometrioid cancer among non-Hispanic White women in all years (APC = −1.3; 95% CI, −1.9 to −0.8), whereas there was a significantly increasing trend among Hispanic women in later years (AAPC during 2010–2019 = 3.6; 95% CI, 1.0 to 6.3; Fig. 1; Table 2). Although all racial/ethnic groups had an increasing trend for clear cell cancer, the results were only significant for Hispanic (APC = 2.8; 95% CI, 0.8 to 4.7) and Asian/Pacific Islander women (APC = 1.5; 95% CI, 0.7 to 2.2; Fig. 1; Table 2).

**TABLE 2:** Log-transformed Joinpoint trends in ovarian cancer incidence by histotype and race/ethnicity, SEER-12, 1992–2019

		Trend 1		Trend 2		Trend 3		2015–2019 AAPC (95% CI)	2010–2019 AAPC (95% CI)
Histotype	Race/ethnicity	Years	APC (95% CI)	Years	APC (95% CI)	Years	APC (95% CI)		
High-grade serous	Asian/Pacific Islander	1992–2019	−1.1 (−1.8 to −0.5)^a^					−1.1 (−1.8 to −0.5)^a^	−1.1 (−1.8 to −0.5)^a^
	Hispanic	1992–2019	−0.7 (−1.2 to −0.1)^a^					−0.7 (−1.2 to −0.1)^a^	−0.7 (−1.2 to −0.1)^a^
	Non-Hispanic Black	1992–2019	−1.1 (−1.9 to −0.2)^a^					−1.1 (−1.9 to −0.2)^a^	−1.1 (−1.9 to −0.2)^a^
	Non-Hispanic White	1992–1998	0.8 (−1.8 to 3.4)	1998–2015	−2.1 (−2.7 to −1.5)^a^	2015–2019	−10.9 (−15.1 to −6.5)^a^	−10.9 (−15.1 to −6.5)^a^	−6.1 (−8.0 to −4.2)^a^
Low-grade endometrioid	Asian/Pacific Islander	1992–2019	0.4 (−0.8 to 1.6)					0.4 (−0.8 to 1.6)	0.4 (−0.8 to 1.6)
	Hispanic	1992–1998	12.7 (0.8 to 26.0)^a^	1998–2003	−12.6 (−29.0 to 7.7)	2003–2019	3.6 (1.0 to 6.3)^a^	3.6 (1.0 to 6.3)^a^	3.6 (1.0 to 6.3)^a^
	Non-Hispanic Black	1992–2019	−1.2 (−3.6 to 1.2)					−1.2 (−3.6 to 1.2)	−1.2 (−3.6 to 1.2)
	Non-Hispanic White	1992–2019	−1.3 (−1.9 to −0.8)^a^					−1.3 (−1.9 to −0.8)^a^	−1.3 (−1.9 to −0.8)^a^
Clear cell	Asian/Pacific Islander	1992–2019	1.5 (0.7 to 2.2)^a^					1.5 (0.7 to 2.2)^a^	1.5 (0.7 to 2.2)^a^
	Hispanic	1992–2019	2.8 (0.8 to 4.7)^a^					2.8 (0.8 to 4.7)^a^	2.8 (0.8 to 4.7)^a^
	Non-Hispanic Black	1992–2019	1.3 (−1.1 to 3.7)					1.3 (−1.1 to 3.7)	1.3 (−1.1 to 3.7)
	Non-Hispanic White	1992–2019	0.3 (−0.3 to 1.0)					0.3 (−0.3 to 1.0)	0.3 (−0.3 to 1.0)

^a^Statistically significant (*P* < 0.05).

### Age-period-cohort Analyses

The high-grade serous cancer incidence increased until ages 60–65 years and then decreased with slightly different peaks depending on racial/ethnic groups; for example, the peak for non-Hispanic Black women came about 10 years later compared with non-Hispanic White women (Supplementary Fig. S2). The incidence of low-grade endometroid and clear cell cancers peaked at ages 45–50 and 55–60, respectively (Supplementary Fig. S2). This indicates that high-grade serous cancers occur later in life compared with low-grade endometrioid and clear cell cancers.

AICs for the age-period-cohort models were not different from the age-cohort models for most histotype and racial/ethnic categories (except the high-grade serous cancer in non-Hispanic White women; Supplementary Table S2), indicating that adding period effects on top of age and cohort effects did not contribute much explaining the trend. While there was no clear trend by period for high-grade serous cancer among Hispanic women, there was a decreasing trend by period for other racial/ethnic groups (Supplementary Fig. S3).

**FIGURE 2:**
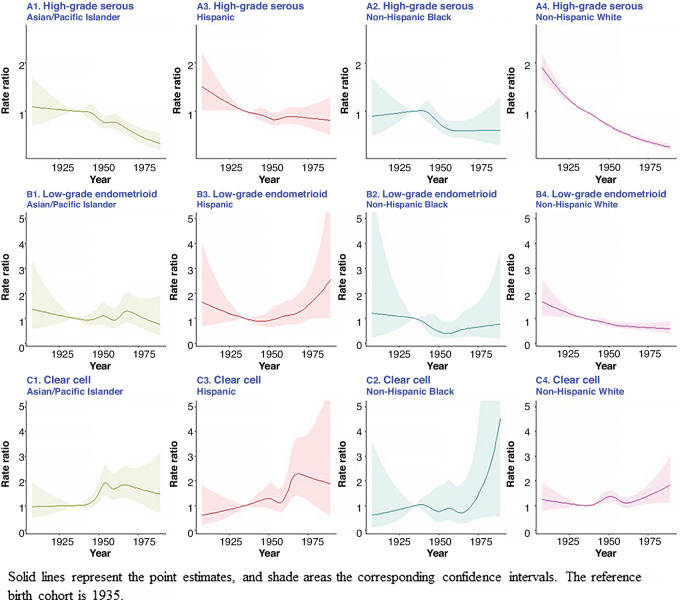
Cohort effects from the age-cohort models, SEER-12, 1992–2019.

Among non-Hispanic White and Asian/Pacific Islander women, there was a decreasing trend by birth cohort for high-grade serous cancer (Fig. 2). The incidence of low-grade endometrioid cancer was decreasing for non-Hispanic White women but increasing in recent birth cohorts among Hispanic women though the CIs were wide. Among non-Hispanic Black women, the trend was relatively constant for high-grade serous and low-grade endometrioid cancers, but the CIs were wide. The wide CIs were possibly due to small sample sizes. In general, the results suggested that clear cell cancer incidence has been increasing by birth cohort. There was a bump in incidence for the 1950–1955 birth cohort for clear cell cancer among all racial/ethnic groups and, more generally, in recent birth cohorts for non-Hispanic White and Black women.

Sensitivity analyses exploring the inclusion of advanced-stage serous cancers missing grade did not materially affect the results presented above. Sensitivity analyses excluding high-grade endometrioid cancer from high-grade serous cancer yielded similar results (Supplementary Fig. S4). Furthermore, using SEER-17 data, which includes more registries, but a shorter time frame compared with SEER-12, provided similar findings from those using SEER-12.

## Discussion

We analyzed trends in ovarian cancer incidence in the United States by histotype and race/ethnicity using SEER-12 registry 1992–2019. The study showed that while high-grade serous cancer is the most common histotype among all racial/ethnic groups, its incidence has been decreasing over time. Conversely, clear cell cancers have been increasing over time and are more common in Asian/Pacific Islander women than in other racial/ethnic groups. Low-grade endometrioid cancer incidence has decreased in non-Hispanic White women but increased in Hispanic women. Our results were similar to a previous analysis of ovarian cancer trends by histotype and race/ethnicity using SEER-18 data during 2000–2013 (30). However, we examined the trends over an extended period and used more updated data (1992–2019) compared with the previous analysis. We also applied Joinpoint and age-period-cohort analyses to examine the trends as well as the period and birth cohort effects on the trends. The changes in incidence may be due to temporal changes in risk and protective factors for these cancers but may also reflect improvement in histotype classification.

In our analyses, we included high-grade endometrioid cancer into high-grade serous cancer, as suggested by the literature. It is evident that high-grade endometrioid ovarian cancers show different gene profiles from low-grade endometrioid tumors but similar gene profiles as high-grade serous tumors (31). In addition, high-grade serous carcinoma may be misclassified as high-grade endometrioid cancer. Gilks and colleagues reviewed 176 tumors originally classified as endometrioid invasive epithelial ovarian cancer and reclassified 50 cases as serous tumors, of which 49 were high-grade serous tumors (26). The authors also looked at the expression of WT-1, which is supposed to be predominantly positive among serous tumors but mostly negative among endometrioid tumors. WT-1 immunostaining supported the reclassification because 68.2% of those 50 tumors were WT-1 positive, which is similar to the proportion of WT-1 positivity in the group of serous carcinomas (77.8%) but is remarkably higher than the proportion of WT-1 positivity in the endometrioid tumors classified by this review (3.9%; refs. 26). These references suggest that there are etiologic similarities between high-grade endometrioid and high-grade serous tumors, and thus it is appropriate to group them. We conducted sensitivity analyses excluding high-grade endometrioid tumors from high-grade serous tumors and found similar results.

The underlying reasons for the decrease in the incidence of advanced-stage, high-grade serous cancer in all racial/ethnic groups over the past 20 years are likely multifaceted. First, estrogen-alone menopausal hormonal therapy (ET) use is associated with an increased risk of high-grade serous cancer (14, 15). In 2002, the prevalence of ET use decreased dramatically following the WHI results linking menopausal estrogen plus progestin (EPT) use with increased risk of cardiovascular heart disease, stroke, and invasive breast cancer (16). Although the WHI result was specific to EPT, both ET and EPT use declined sharply in the United States (32) and has continued to decrease at least through 2013 (33). Use of ET is not associated with the risk of clear cell ovarian cancer (14), consistent with our observation that the incidence of this histotype is not decreasing but rather increasing.

Another driver for the decreasing trend in high-grade serous cancer incidence could be the increase in opportunistic salpingectomy, which is the removal of the fallopian tubes during abdominal surgery or in lieu of tubal ligation. High-grade serous cancer is thought to arise from the fallopian tube fimbriae (17); thus, salpingectomy is recommended as a preventive strategy for high-grade serous cancer (34–37). The number of salpingectomies in the United States increased in 2008 and during the 2011–2013 period went up by 71% (38). This coincided with the sharp decline in the number of high-grade serous cases from 2011 to 2018, especially from 2014 to 2018 (24%). However, neither the decrease in ET use nor the increased uptake of opportunistic salpingectomy would fully explain the declining high-grade serous incidence we observed in the late 1990s.

We also found an increasing trend in clear cell cancer incidence for all racial/ethnic groups, especially among Asian/Pacific Islanders. This may also be explained by improvement in the diagnosis of this histotype. There was a pattern of shifting away from classifying tumors as “carcinoma, not otherwise specified” (NOS) to clear cell carcinoma in all racial/ethnic groups, but most striking in Asian/Pacific Islanders. Using SEER-12 registry data, we found that during 1992–2019, the proportion of NOS among Asian/Pacific Islanders decreased from 20% to 11%, while the proportion of clear cell cancer increased by the same amount, from 9% to 18% (Supplementary Fig. S5). This pattern was less obvious among other racial/ethnic groups, in which the increase of clear cell carcinoma proportion was smaller than the decrease in carcinoma NOS (Hispanics: clear cell increased by 5% while carcinoma NOS decreased by 9%, non-Hispanic Black: 1% vs. 2%; non-Hispanic White 5% vs. 10%; Supplementary Fig. S5). The reason for such a striking shift in diagnosis among Asian/Pacific Islanders could be an increase in awareness among pathologists, given that recent studies have found that clear cell cancer is much more common in Asian countries. Clear cell cancer accounted for about 20% of all epithelial ovarian cancer cases in Singapore and Japan during 2003–2007 (4), which was much higher than the proportion in the United States at 10% during 2004–2014 (24).

Previous temporal trends in declining ovarian cancer incidence in the 1970s and 1980s were attributed to increasing parity and combined oral contraceptive use, both protective factors for all ovarian cancer histotypes. However, more recently, combined oral contraceptive use prevalence has decreased in non-Hispanic Black and Hispanic women and the fertility rate in all racial/ethnic groups has been decreasing. The impact of such decreases may differ by histotype as the magnitude of the associations for high-grade serous cancer is weaker than one for clear cell cancers (odds ratio (OR) for one birth vs. nulliparous: 0.89 and 0.39 for serous and clear cell cancer, respectively; OR for 2–4.99 years of combined oral contraceptive use vs. never use: 0.65 and 0.55 for serous and clear cell cancers, respectively; ref. 9). Therefore, the impact of the decline in parity and combined oral contraceptive use on high-grade serous incidence would likely be less than one on clear cell cancer. The observation that the endometrioid cancer incidence decreased in non-Hispanic White but increased in Hispanic women in recent years may be partially explained by the racial/ethnic differences in the decline in parity and combined oral contraceptive use, given the strength of these associations (39, 40). Also, the increase in clear cell rates may be due to the changes in parity and combined oral contraceptive use.

Two additional interesting observations for birth cohort effects are the small but noticeable peak around birth cohorts 1950–1955 for clear cell cancer in all racial/ethnic groups and that the peak for high-grade serous cancer incidence is about 10 years later in non-Hispanic Black women compared with non-Hispanic White women. The peak in clear cell cancer incidence might partially be explained by the exposure to diethylstilbestrol (DES), a potent synthetic estrogen, which was given to pregnant women in the United States from the early 1940s through the 1960s for the prevention of spontaneous abortion and premature delivery (41). Use of DES in pregnancy was discontinued in 1971 when there was evidence of a strong association between prenatal exposure to DES and an elevated risk of clear cell adenocarcinoma of the vagina and cervix (42). There is some evidence that an increased risk of clear cell ovarian cancer may be associated with prenatal DES exposure (43), although population studies did not find an association, possibly due to small sample sizes (44–46). The 10-year later peak in high-grade serous incidence for non-Hispanic Black women is likely due to a large number of hysterectomies (likely with an oophorectomy) that occurred in this population (47). Generally, it is likely that the population at risk denominator for calculations for ovarian cancer incidence for non-Hispanic Black women is inflated because of the high prevalence of hysterectomy with oophorectomy.

There are some limitations of the study. First, the ecologic nature of the study does not allow for causal inferences. The SEER data do not contain information on risk factors for ovarian cancer. Second, U.S.-born and foreign-born women may have different cohort effects, but we could not examine the difference because the SEER registry removed the country of birth variable in recent submissions due to bias (personal communication with the SEER support team). Third, there might be a risk of misclassifying ovarian cancer histotypes across the registries. To minimize this risk, we conducted a sensitivity analysis including patients with advanced-stage serous cancer (distant or regional stage) with a missing grade as high-grade serous cases. Despite these limitations, the SEER data used in our study are the gold standard for cancer registration (48).

In conclusion, this study provides the most current overview of ovarian cancer trends by histotype and race/ethnicity in the United States and examines the influence of period and birth cohort on the trends. We found that high-grade serous cancer incidence has decreased overall, while clear cell cancer incidence has increased. The differences in the trends by histotype and race/ethnicity can partially be explained by the variations in the prevalence of risk factors by year and cohort across race/ethnicity and their different effect magnitudes by histotype, as well as the changes in diagnostic classification. It is crucial to develop targeted preventive interventions to effectively reduce the burden of ovarian cancer and disparity by race/ethnicity. There is a need to continue monitoring the incidence trends of ovarian cancer, particularly clear cell cancer to understand whether the increase in incidence was due to changes in risk factor prevalence or the shift in histotype classification. In addition, it is important to keep monitoring the incidence trends of ovarian cancer by race/ethnicity to develop targeted prevention strategies.

## Supplementary Material

Supplementary Table ST1 Supplementary Table 1 shows Registries included in SEER-12 and SEER-17

Supplementary Table ST2 Supplementary Table 2 shows Akaike Information Criteria (AIC) for Age-Cohort models and Age-Period-Cohort models and their differences, SEER-12, 1992-2019

Supplementary Figure S1 Supplementary Figure 1 shows the flow chart of case exclusion for analysis, SEER-12, 1992-2019

Supplementary Figure S2 Supplementary Figure 2 shows age effects in the age-cohort models, SEER-12, 1992-2019

Supplementary Figure S3 Supplementary Figure 3 shows period effects in the age-period models, SEER-12, 1992-2019

Supplementary Figure S4 Supplementary Figure 4 shows age-standardized incidence rate (per 1,000,000) of high-grade serous ovarian cancer by race/ethnicity. The incidence in the left panel did not include high-grade endometrioid cancer. The incidence in the right panel included high-grade endometrioid cancer in high-grade serous cancer.

Supplementary Figure S5 Supplementary Figure 5 shows the distribution of histotype by race/ethnicity, SEER-12, 1992-2019

## References

[1] Siegel RL , Miller KD, Jemal A. Cancer statistics, 2020. CA Cancer J Clin 2020;70:7–30. 31912902 10.3322/caac.21590

[2] Torre LA , Trabert B, DeSantis CE, Miller KD, Samimi G, Runowicz CD, . Ovarian cancer statistics, 2018. CA Cancer J Clin 2018;68:284–96. 29809280 10.3322/caac.21456 PMC6621554

[3] American Cancer Society. Cancer facts & figures 2018; 2018.

[4] Coburn SB , Bray F, Sherman ME, Trabert B. International patterns and trends in ovarian cancer incidence, overall and by histologic subtype. Int J Cancer 2017;140:2451–60. 28257597 10.1002/ijc.30676 PMC5595147

[5] Lima SM , Kehm RD, Swett K, Gonsalves L, Terry MB. Trends in parity and breast cancer incidence in US women younger than 40 years from 1935 to 2015. JAMA Netw Open 2020;3:e200929. 32167569 10.1001/jamanetworkopen.2020.0929 PMC7070232

[6] Whittemore AS , Balise RR, Pharoah PD, Dicioccio RA, Oakley-Girvan I, Ramus SJ, . Oral contraceptive use and ovarian cancer risk among carriers of BRCA1 or BRCA2 mutations. Br J Cancer 2004;91:1911–5. 15545966 10.1038/sj.bjc.6602239 PMC2410144

[7] Pike MC , Pearce CL, Peters R, Cozen W, Wan P, Wu AH. Hormonal factors and the risk of invasive ovarian cancer: a population-based case-control study. Fertil Steril 2004;82:186–95. 15237010 10.1016/j.fertnstert.2004.03.013

[8] Wentzensen N , Poole EM, Trabert B, White E, Arslan AA, Patel AV, . Ovarian cancer risk factors by histologic subtype: an analysis from the ovarian cancer cohort consortium. J Clin Oncol 2016;34:2888–98. 27325851 10.1200/JCO.2016.66.8178 PMC5012665

[9] Pearce CL , Rossing MA, Lee AW, Ness RB, Webb PM, Chenevix-Trench G, . Combined and interactive effects of environmental and GWAS–identified risk factors in ovarian cancer. Cancer Epidemiol Biomarkers Prev 2013;22:880–90. 23462924 10.1158/1055-9965.EPI-12-1030-T PMC3963289

[10] Kim SJ , Rosen B, Fan I, Ivanova A, McLaughlin JR, Risch H, . Epidemiologic factors that predict long-term survival following a diagnosis of epithelial ovarian cancer. Br J Cancer 2017;116:964–71. 28208158 10.1038/bjc.2017.35 PMC5379147

[11] Kvåle G , Heuch I, Nilssen S, Beral V. Reproductive factors and risk of ovarian cancer: a prospective study. Int J Cancer 1988;42:246–51. 3403067 10.1002/ijc.2910420217

[12] Hankinson SE , Colditz GA, Hunter DJ, Willett WC, Stampfer MJ, Rosner B, . A prospective study of reproductive factors and risk of epithelial ovarian cancer. Cancer 1995;76:284–90. 8625104 10.1002/1097-0142(19950715)76:2<284::aid-cncr2820760219>3.0.co;2-5

[13] King LA , Michels KA, Graubard BI, Trabert B. Trends in oral contraceptive and intrauterine device use among reproductive-aged women in the US from 1999 to 2017. Cancer Causes Control 2021;32:587–95. 33689082 10.1007/s10552-021-01410-8 PMC8096680

[14] Lee AW , Ness RB, Roman LD, Terry KL, Schildkraut JM, Chang-Claude J, . Association between menopausal estrogen-only therapy and ovarian carcinoma risk. Obstet Gynecol 2016;127:828–36. 27054934 10.1097/AOG.0000000000001387 PMC4892111

[15] Beral V , Gaitskell K, Hermon C, Moser K, Reeves G, Peto R, . Menopausal hormone use and ovarian cancer risk: individual participant meta-analysis of 52 epidemiological studies. Lancet 2015;385:1835–42. 25684585 10.1016/S0140-6736(14)61687-1 PMC4427760

[16] Rossouw JE , Anderson GL, Prentice RL, LaCroix AZ, Kooperberg C, Stefanick ML, . Risks and benefits of estrogen plus progestin in healthy postmenopausal women: principal results from the Women's Health Initiative randomized controlled trial. JAMA 2002;288:321–33. 12117397 10.1001/jama.288.3.321

[17] Karnezis AN , Cho KR, Gilks CB, Pearce CL, Huntsman DG. The disparate origins of ovarian cancers: pathogenesis and prevention strategies. Nat Rev Cancer 2017;17:65–74. 27885265 10.1038/nrc.2016.113

[18] Wu AH , Pearce CL, Tseng CC, Pike MC. African americans and hispanics remain at lower risk of ovarian cancer than non-hispanic whites after considering nongenetic risk factors and oophorectomy rates. Cancer Epidemiol Biomarkers Prev 2015;24:1094–100. 25873577 10.1158/1055-9965.EPI-15-0023 PMC4490941

[19] Webb PM , Green AC, Jordan SJ. Trends in hormone use and ovarian cancer incidence in US white and Australian women: implications for the future. Cancer Causes Control 2017;28:365–70. 28233113 10.1007/s10552-017-0868-0

[20] Mink PJ , Sherman ME, Devesa SS. Incidence patterns of invasive and borderline ovarian tumors among white women and black women in the United States. Results from the SEER Program, 1978–1998. Cancer 2002;95:2380–9. 12436446 10.1002/cncr.10935

[21] Cramer DW , Devesa SS, Welch WR. Trends in the incidence of endometrioid and clear cell cancers of the ovary in the United States. Am J Epidemiol 1981;114:201–8. 7304555 10.1093/oxfordjournals.aje.a113183

[22] NCI. Surveillance, Epidemiology, and End Results Program. Available from: https://seer.cancer.gov/.

[23] Surveillance Epidemiology, and End Results (SEER) Program (www.seer.cancer.gov) SEER*Stat Database,. Incidence – SEER Research Data, 12 Registries, Nov 2021 Sub (1992–2019) – Linked To County Attributes – Time Dependent (1990–2019) Income/Rurality, 1969–2020 Counties. National Cancer Institute D, Surveillance Research Program, editorreleased April 2022, based on the November 2021 submission.

[24] Peres LC , Cushing-Haugen KL, Köbel M, Harris HR, Berchuck A, Rossing MA, . Invasive epithelial ovarian cancer survival by histotype and disease stage. J Natl Cancer Inst 2019;111:60–8. 29718305 10.1093/jnci/djy071 PMC6335112

[25] Seidman JD , Horkayne-Szakaly I, Haiba M, Boice CR, Kurman RJ, Ronnett BM. The histologic type and stage distribution of ovarian carcinomas of surface epithelial origin. Int J Gynecol Pathol 2004;23:41–4. 14668549 10.1097/01.pgp.0000101080.35393.16

[26] Gilks CB , Ionescu DN, Kalloger SE, Köbel M, Irving J, Clarke B, . Tumor cell type can be reproducibly diagnosed and is of independent prognostic significance in patients with maximally debulked ovarian carcinoma. Hum Pathol 2008;39:1239–51. 18602670 10.1016/j.humpath.2008.01.003

[27] Hoyert DL , Anderson RN. Age-adjusted death rates: trend data based on the year 2000 standard population. Natl Vital Stat Rep 2001;49:1–6. 11589033

[28] NCI. Joinpoint trend analysis software. 4.9.1.02022.

[29] Kim HJ , Fay MP, Feuer EJ, Midthune DN. Permutation tests for joinpoint regression with applications to cancer rates. Stat Med 2000;19:335–51. 10649300 10.1002/(sici)1097-0258(20000215)19:3<335::aid-sim336>3.0.co;2-z

[30] Park HK , Ruterbusch JJ, Cote ML. Recent trends in ovarian cancer incidence and relative survival in the United States by race/ethnicity and histologic subtypes. Cancer Epidemiol Biomarkers Prev 2017;26:1511–8. 28751475 10.1158/1055-9965.EPI-17-0290 PMC6859937

[31] Schwartz DR , Kardia SL, Shedden KA, Kuick R, Michailidis G, Taylor JM, . Gene expression in ovarian cancer reflects both morphology and biological behavior, distinguishing clear cell from other poor-prognosis ovarian carcinomas. Cancer Res 2002;62:4722–9. 12183431

[32] Hersh AL , Stefanick ML, Stafford RS. National use of postmenopausal hormone therapy: annual trends and response to recent evidence. JAMA 2004;291:47–53. 14709575 10.1001/jama.291.1.47

[33] Crawford SL , Crandall CJ, Derby CA, El Khoudary SR, Waetjen LE, Fischer M, . Menopausal hormone therapy trends before versus after 2002: impact of the Women's Health Initiative Study results. Menopause 2019;26:588–97. 10.1097/GME.0000000000001282 PMC6538484 30586004

[34] Dilley SE , Straughn JM, Leath CA. The evolution of and evidence for opportunistic salpingectomy. Obstet Gynecol 2017;130:814–24. 28885426 10.1097/AOG.0000000000002243

[35] Society of Gynecologic Oncology of Canada. GOC Statement regarding salpingectomy and ovarian cancer prevention. 2011;2017.

[36] Society of Gynecologic Oncology. SGO clinical practice statement: salpingectomy for ovarian cancer prevention. SGO Chicago; 2013.

[37] Committee opinion no. 620: salpingectomy for ovarian cancer prevention. Obstet Gynecol 2015;125:279–81. 25560145 10.1097/01.AOG.0000459871.88564.09

[38] Hicks-Courant KD . Growth in salpingectomy rates in the United States since. Am J Obstet Gynecol 2016;215:666–7. 27498310 10.1016/j.ajog.2016.07.055

[39] National Center for Health Statistics. National Survey of Family Growth, 2006–2010, 2011–2013, 2013–2015, 2015–2017, 2017–2019.

[40] National Center for Health Statistics. National vital statistics, 1989–2018.

[41] Noller KL , Fish CR. Diethylstilbestrol usage: its interesting past, important present, and questionable future. Med Clin North Am 1974;58:793–810. 4276416 10.1016/s0025-7125(16)32122-8

[42] Herbst AL , Ulfelder H, Poskanzer DC. Adenocarcinoma of the vagina. Association of maternal stilbestrol therapy with tumor appearance in young women. N Engl J Med 1971;284:878–81. 5549830 10.1056/NEJM197104222841604

[43] Dasanu CA , Herzog TJ. Clear cell adenocarcinoma of the ovary associated with *in utero* diethylstilbestrol exposure: case report and clinical overview. Medscape J Med 2009;11:6. 19295927 PMC2654676

[44] Titus-Ernstoff L , Hatch EE, Hoover RN, Palmer J, Greenberg ER, Ricker W, . Long-term cancer risk in women given diethylstilbestrol (DES) during pregnancy. Br J Cancer 2001;84:126–33. 11139327 10.1054/bjoc.2000.1521 PMC2363605

[45] Hatch EE , Palmer JR, Titus-Ernstoff L, Noller KL, Kaufman RH, Mittendorf R, . Cancer risk in women exposed to diethylstilbestrol *in utero*. JAMA 1998;280:630–4. 9718055 10.1001/jama.280.7.630

[46] Troisi R , Hatch EE, Titus L, Strohsnitter W, Gail MH, Huo D, . Prenatal diethylstilbestrol exposure and cancer risk in women. Environ Mol Mutagen 2019;60:395–403. 29124779 10.1002/em.22155

[47] Jamison PM , Noone AM, Ries LA, Lee NC, Edwards BK. Trends in endometrial cancer incidence by race and histology with a correction for the prevalence of hysterectomy, SEER 1992 to 2008. Cancer Epidemiol Biomarkers Prev 2013;22:233–41. 23239812 10.1158/1055-9965.EPI-12-0996

[48] Duggan MA , Anderson WF, Altekruse S, Penberthy L, Sherman ME. The surveillance, epidemiology, and end results (SEER) program and pathology: toward strengthening the critical relationship. Am J Surg Pathol 2016;40:e94–e102. 27740970 10.1097/PAS.0000000000000749 PMC5106320

