# Supplementary material for: Trends of Ovarian Cancer Incidence by Histotype and Race/Ethnicity in the United States 1992–2019
Source: Cancer Res Commun. 2023 Jan 3;3(1):1–8. doi: 10.1158/2767-9764.CRC-22-0410 (PMC10035532; doi:10.1158/2767-9764.CRC-22-0410)
Supplement: Supplementary Table ST1 — Supplementary Table 1 shows Registries included in SEER-12 and SEER-17 [file crc-22-0410-s01.docx]

| **Supplementary Table 1: Registries included in SEER-12 and SEER-17** | | |
| --- | --- | --- |
| SEER-12 included cases diagnosed from 12 registries during 1992-2019. | | |
| SEER-17 included cases diagnosed from 17 registries during 2000-2019. | | |
| Source: https://seer.cancer.gov/registries/terms.html#footnoteb | | |
|  |  |  |
| **Registry** | **SEER-12** | **SEER-17** |
| Alaska Native Tumor Registry | X | X |
| Connecticut | X | X |
| Atlanta | X | X |
| Greater Georgia |  | X |
| Rural Georgia | X | X |
| San Francisco-Oakland | X | X |
| San Jose-Monterey | X | X |
| Greater California |  | X |
| Hawaii | X | X |
| Iowa | X | X |
| Kentucky |  | X |
| Los Angeles | X | X |
| Louisiana |  | X |
| New Mexico | X | X |
| New Jersey |  | X |
| Seattle-Puget Sound | X | X |
| Utah | X | X |
