# Supplementary material for: Trends of Ovarian Cancer Incidence by Histotype and Race/Ethnicity in the United States 1992–2019
Source: Cancer Res Commun. 2023 Jan 3;3(1):1–8. doi: 10.1158/2767-9764.CRC-22-0410 (PMC10035532; doi:10.1158/2767-9764.CRC-22-0410)
Supplement: Supplementary Table ST2 — Supplementary Table 2 shows Akaike Information Criteria (AIC) for Age-Cohort models and Age-Period-Cohort models and their differences, SEER-12, 1992-2019 [file crc-22-0410-s02.docx]

## **Supplementary Table 2: Akaike Information Criteria (AIC) for Age-Cohort models and Age-Period-Cohort models and their differences, SEER-12, 1992-2019**

| **Histotype** | **Race/ethnicity** | **AIC Age-Cohort Model** | **AIC Age-Period-Cohort Model** | **Difference in AIC between the Age-Period-Cohort Model and Age-Cohort Model^a^** |
| --- | --- | --- | --- | --- |
| High-grade serous | Asian/Pacific Islander | 1631.0 | 1621.9 | 0.8 |
|  | Hispanic | 1682.8 | 1674.4 | 1.6 |
|  | Non-Hispanic Black | 1498.6 | 1494.1 | 5.5 |
|  | Non-Hispanic White | 1692.8 | 1597.6 | -85.2 |
| Low-grade endometrioid | Asian/Pacific Islander | 1153.7 | 1151.7 | 8.0 |
|  | Hispanic | 1067.4 | 1065.9 | 8.5 |
|  | Non-Hispanic Black | 638.1 | 637.1 | 9.0 |
|  | Non-Hispanic White | 1660.1 | 1655.9 | 5.7 |
| Clear cell | Asian/Pacific Islander | 1287.0 | 1282.8 | 5.8 |
|  | Hispanic | 1036.4 | 1034.2 | 7.7 |
|  | Non-Hispanic Black | 540.0 | 536.2 | 6.3 |
|  | Non-Hispanic White | 1635.2 | 1632.3 | 7.0 |

**^a^** Relative that weight the goodness of fit of the model to the Age-Cohort model.

**^b^**AIC is computed by -2×log(likelihood)+2×number of estimated parameters. The lower the AIC, the better the model fit.
