# Supplementary material for: Trends of Ovarian Cancer Incidence by Histotype and Race/Ethnicity in the United States 1992–2019
Source: Cancer Res Commun. 2023 Jan 3;3(1):1–8. doi: 10.1158/2767-9764.CRC-22-0410 (PMC10035532; doi:10.1158/2767-9764.CRC-22-0410)
Supplement: Supplementary Figure S1 — Supplementary Figure 1 shows the flow chart of case exclusion for analysis, SEER-12, 1992-2019 [file crc-22-0410-s03.docx]

## **Supplementary Figure 1. Flow chart of case exclusion for analysis, SEER-12, 1992-2019**

Microscopically confirmed invasive ovarian cancer diagnosed between 1992-2019 in
SEER-12 (N=55,106)

Non-epithelial or other miscellaneous tumors (n=4,786)

Epithelial ovarian cancer (N=50,320)

Age of diagnosis ≥85 years (n=2,465) or <30 years (n=748)

Age of diagnosis, 30-84 years (N=47,107)

Unknown race/ethnicity (n=121)

American Indian/Alaska Native (n=458)

Included race/ethnicity (N=46,528)

**High-grade serous (n=19,691)**

**Low-grade endometrioid (n=3,212)**

**Clear cell (n=3,275)**

Serous missing grade (n=5,313)

Endometrioid missing grade (n=582)

Low-grade serous (n=787)

Mucinous (n=3,202)

Carcinosarcoma (n=1,430)

Malignant Brenner (n=110)

Carcinoma, NOS (n=7,330)

Mixed (n=1,596)
