# Supplementary figures and images for: Trends of Ovarian Cancer Incidence by Histotype and Race/Ethnicity in the United States 1992–2019
Source: Cancer Res Commun. 2023 Jan 3;3(1):1–8. doi: 10.1158/2767-9764.CRC-22-0410 (PMC10035532; doi:10.1158/2767-9764.CRC-22-0410)

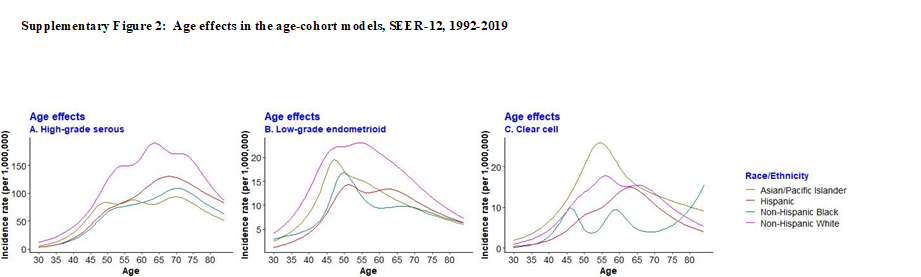

Supplement: Supplementary Figure S2 — Supplementary Figure 2 shows age effects in the age-cohort models, SEER-12, 1992-2019 [file crc-22-0410-s04.png]

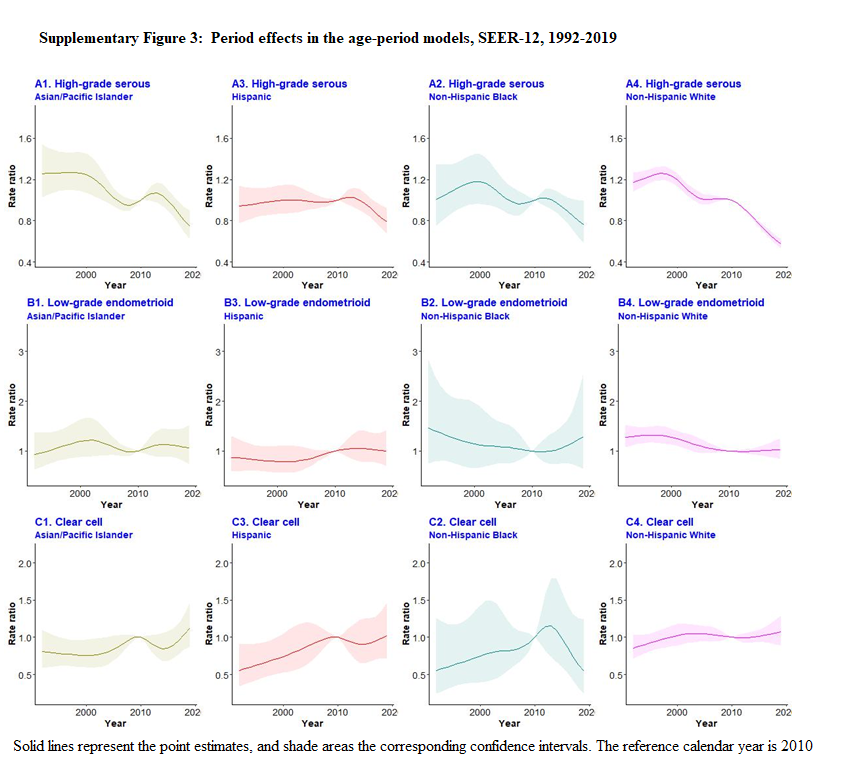

Supplement: Supplementary Figure S3 — Supplementary Figure 3 shows period effects in the age-period models, SEER-12, 1992-2019 [file crc-22-0410-s05.png]

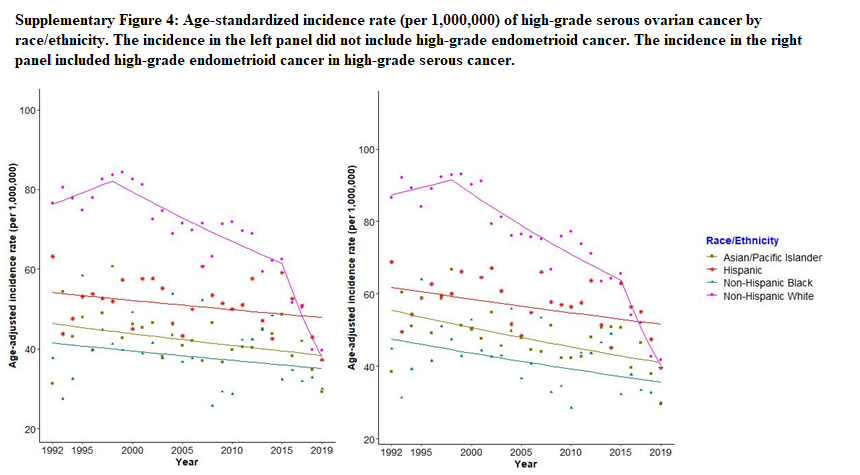

Supplement: Supplementary Figure S4 — Supplementary Figure 4 shows age-standardized incidence rate (per 1,000,000) of high-grade serous ovarian cancer by race/ethnicity. The incidence in the left panel did not include high-grade endometrioid cancer. The incidence in the right panel included high-grade endometrioid cancer in high-grade serous cancer. [file crc-22-0410-s06.png]

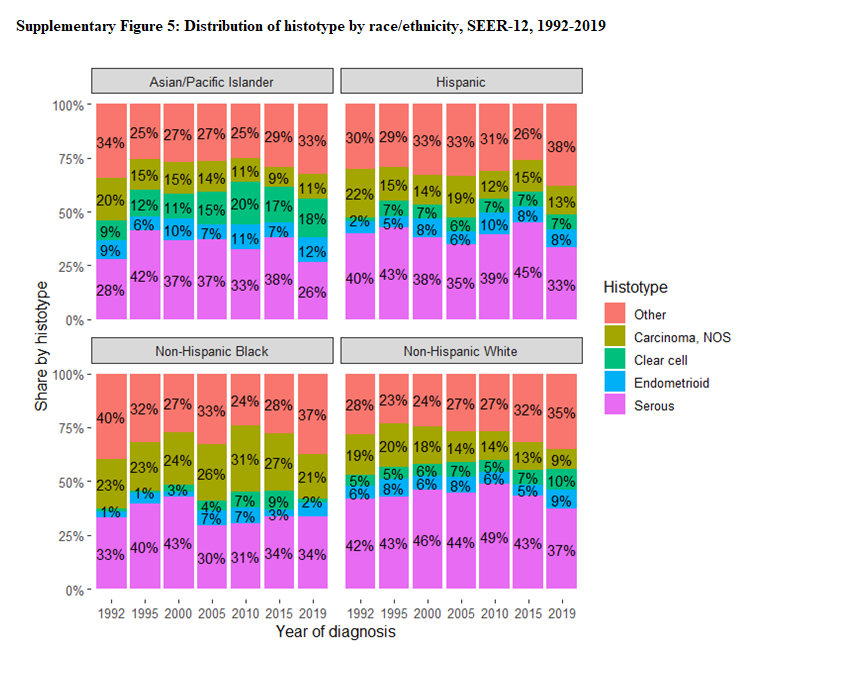

Supplement: Supplementary Figure S5 — Supplementary Figure 5 shows the distribution of histotype by race/ethnicity, SEER-12, 1992-2019 [file crc-22-0410-s07.png]
